# The Association between Dry Mouth and the Periodontal Status in Older Adults Undergoing Supportive Periodontal Therapy

**DOI:** 10.1055/s-0045-1809183

**Published:** 2025-05-21

**Authors:** Pasiri Tangsumroengvong, Kununya Pimolbutr, Nataya Nopparattanakant, Pimsunee Lowpradit, Kallapat Tansriratanawong

**Affiliations:** 1 Department of Geriatric Dentistry, Faculty of Dentistry, Mahidol University, Bangkok, Thailand; 2 Department of Oral Medicine and Periodontology, Faculty of Dentistry, Mahidol University, Bangkok, Thailand

**Keywords:** age-related changes, aging, dry mouth, supportive periodontal treatment, recurrent periodontitis

## Abstract

**Objective:**

Dry mouth is a common oral condition in older adults, which correlates with dehydration, pH changes, and lubrication in the oral cavity, leading to an imbalance among bacterial activities of dental biofilms. However, the role of dry mouth and periodontal status in older adults has remained limited, especially among periodontal individuals who underwent supportive periodontal therapy (SPT). This study aimed to investigate the association between dry mouth and recurrent periodontitis in older adults undergoing SPT.

**Materials and Methods:**

This cross-sectional study included patients who were part of SPT. The factors of interest were collected by interviews, questionnaires, and clinical assessments. Oral moisture measurement, the clinical oral dryness score, unstimulated salivary flow rate (USSFR), and stimulated salivary flow rate (SSFR) were performed. Full mouth periodontal examination was performed and compared with the previous record of periodontal examination to identify a recurrence of periodontitis based on bleeding on probing (BOP), a change in pocket depth, and clinical attachment level. Descriptive analysis, both univariate and multivariate logistic regression, was performed to delineate the association between dry mouth and recurrent periodontitis.

**Results:**

A total of 186 participants were recruited and divided into the recurrent periodontitis ( *n* = 37) and the nonrecurrent periodontitis group ( *n* = 149). Baseline demographics, medical and dental history of the two groups were similar. Participants with hyposalivation were greater in the recurrent group (35.1 vs. 16.1%, *p* = 0.02), and the mean of USSFR is lower than the nonrecurrent periodontitis group (0.38 vs. 0.53 mL/min, *p* = 0.01). To examine the relationship between various factors affecting the recurrent periodontitis by using multivariate regression analysis, results demonstrated odds ratio (OR) of hyposalivation and percentage of BOP (%BOP) in recurrent periodontitis at 2.63 (95% CI = 1.05–6.58), *p* = 0.04 and 1.04 (95% CI = 1.02–1.06), *p* < 0.001 after adjusting for confounding factors.

**Conclusions:**

This study supported the hypothesis that hyposalivation is associated with recurrent periodontitis demonstrated by USSFR and %BOP association. Consistent periodontal care, including an examination and guidance on managing dry mouth, has the potential to help older individuals with periodontitis maintaining their dental health.

## Introduction

Periodontitis is a chronic inflammatory disease characterized by dysbiosis, which refers to an imbalance in bacterial biofilms and immune responses. This dysbiosis triggers a heightened proinflammatory reaction in the immune system, leading to damage to the supporting tissues of the teeth. 1 2 Understanding the risks associated with periodontitis is crucial, particularly for older adults, who may be more susceptible due to the cumulative effects of dysbiosis over time. Previous studies have demonstrated that the prevalence of severe chronic periodontitis increases with age from 15 to 54 years, subsequently exhibiting a gradual decline in older age groups. However, the highest percentage increase in the number of prevalent cases was observed in the population aged 70 to 74 years. 3 Numerous theories concerning the aging process suggest that it is linked to a variety of biological changes. These include the phenomena of oxidative stress, mitochondrial dysfunction, and DNA damage. 4 Moreover, older adults, particularly those with multiple health complications, exhibit an increased risk of developing periodontitis. In addition, environmental and systemic factors such as smoking, diabetes mellitus (DM), and certain medications—specifically antidepressants and antihypertensives—that diminish salivary flow can elevate the risk of imbalances in bacterial species, lubrication, and pH levels within biofilms, ultimately contributing to the dysbiosis. 1 2 5

Active periodontal treatment (APT) focuses on reducing bacterial deposits and managing immune-inflammatory responses. This treatment approach encompasses surgical and nonsurgical procedures aimed at eliminating periodontal etiologies. Achieving successful clinical outcomes is contingent upon effective plaque control and the consistent implementation of supportive periodontal treatment (SPT). 6 SPT is a maintenance program involving periodic exams and preventive care to diagnose and treat new or recurring periodontal diseases early. The evaluation schedule is set after completing the initial APT. The effectiveness of periodontal therapy, regular and long-term SPT in preventing tooth loss for individuals with periodontitis, as well as in improving oral health-related quality of life, has been demonstrated. 6 7 8 Therefore, it is essential to assess patients' risks for the recurrence and progression of periodontitis, and determine the appropriate intervals for SPT within clinical practice. 9

Dry mouth can be characterized through both subjective and objective criteria. Xerostomia refers to the patient-reported sensation of dry mouth, while salivary gland hypofunction denotes an objective reduction in salivary production, which can be assessed by measuring salivary flow rates. 10 There are various predisposing factors for dry mouth, including advancing age, female gender, and the consumption of certain medications that impact salivary secretion. 11 It has been shown that ∼35% of people aged above 65 experience xerostomia. 10 Moreover, dry mouth can increase the risk of mucosal inflammation, mastication, oral candidiasis, dental caries, and periodontal problems. 12 Given the increasing prevalence of dry mouth in the elderly demographic, it is imperative that dental professionals remain vigilant regarding this condition and offer suitable treatment options for older individuals.

A lack of saliva can promote bacteria adhesion to the tooth surfaces and may alter the composition of microbial plaque due to a lack of lubrication and changes in pH. Moreover, protection from saliva is a crucial first line of defense mechanism against pathogens, with disturbances in oral infections due to increasing risk of periodontitis. 10 13 14 Furthermore, a previous study showed that a lower rate of salivary flow may be associated with an increased probability of periodontal disease in elderly people. 15 Research investigating the relationship between dry mouth and periodontal disease is limited, especially in SPT patients. Only one study by Sparrow et al. reported that clinical outcomes of people with or without dry mouth were similar when receiving regular periodontal maintenance after scaling and root planing. 16 There is scarce available information relating to the effect of dry mouth on the periodontal status of the SPT program in elderly people. Therefore, this study aimed to investigate whether there is an association between dry mouth (xerostomia and hyposalivation) and recurrent periodontitis in older adults undergoing SPT.

## Materials and Methods

### Study Population

This cross-sectional study was conducted at the Periodontics Clinic, Faculty of Dentistry and Maha Chakri Sirindhorn Dental Hospital, Mahidol University, Thailand. The study population consisted of participants who routinely received SPT after completing their APT. Participants were recruited during the SPT visit between September 2022 and April 2023. Informed consent forms were completed by all participants prior to participation in the study. The present study was approved by the Institutional Review Board, Faculty of Dentistry/Faculty of Pharmacy, Mahidol University (MU-DT/PY-IRB 2022/035.0308). This study was reported according to the Strengthening the Reporting of Observation Studies in Epidemiology (STROBE) guidelines. 17

Inclusion criteria were the following: patients aged 60 years and older, who were diagnosed with periodontitis stages 3 to 4 according to the American Academy of Periodontology (AAP) and the European Federation of Periodontology (EFP) classification in 2017, 18 underwent APT, controlled systemic diseases with at least 14 teeth in the oral cavity. 7 19 Patients were excluded from the study if they met any of the following reasons: (1) patients who were previously diagnosed with gingivitis, (2) patients with a history of radiotherapy in the head and neck, (3) patients with systemic diseases that affect saliva production (e.g., Sjogren's syndrome, dialyzed patients), (4) patients diagnosed with systemic diseases that affect the ability to maintain oral hygiene (e.g., Alzheimer's disease, Parkinson's disease), (5) patients with uncontrolled systemic, (6) antibiotic prescribed within 30 days prior to the study visit, (7) current use of medications that affect periodontal status, (8) current use of immunosuppressant medications, (9) unstable dose of any medications known to have influence upon saliva production for more than 4 weeks before study visit, (10) patients who received periodontal treatment within the period of suggested SPT interval before starting the study, and (11) patients with any oral mucosal lesions.

The sample size was calculated based on a previous study by Costa et al. reporting the progression proportion of periodontal disease after APT of 32%, 20 considering the effect size of 0.20 (20%), a level of significance of 5% ( *α* = 0.05), and a power of 80% ( *β* = 0.2). Therefore, a total sample of 184 patients was required.

### Data Collection and Questionnaires

All eligible participants were asked to fill out self-reported questionnaires. Data regarding age, gender, height, weight, current medical conditions, regular medications used, smoking status, the use of saliva substitutes, tooth brushing frequency, and interdental cleaning were obtained. The 10-item Barthel Index for Activities of Daily Living was used to assess functional capacity. A total score was calculated with a range of 0 to 20 points. Patients were then categorized into three groups according to the sum score: 0 to 4 (total dependence), 5 to 11 (moderate dependence), and ≥ 12 (independence). 21 In addition, depressive symptoms among older adults were evaluated using the Geriatric Depression Scale-15 (GDS-15, Supplementary Table S1 [available in the online version only]). A total score was calculated and assigned to one of three categories: 0 to 4 (normal), 5 to 9 (suggestive of depression), and ≥ 10 (depression). 22

### Clinical and Periodontal Examination

Both oral and a complete periodontal examination were performed at the study visit. The number of remaining teeth, tooth mobility, the number of occluded teeth, wearing dentures, and the SPT interval between the previous visit and the present study visit were recorded.

Regarding periodontal clinical parameters, the percentage of plaque (%plaque score), percentage of bleeding on probing (%BOP), pocket depth (PD), and clinical attachment level (CAL) were recorded. Recurrent periodontitis was determined by an increase of PD (PD) 3 mm with persistent BOP, and then this was confirmed by the detection of a change in CAL (ΔCAL) ≥ 2 mm between the previous SPT visit and the present study visit by the calibrated examiner. 23 24 Participants were then allocated into two groups according to their periodontal status: recurrent periodontitis and nonrecurrent periodontitis.

### Self-Reported Xerostomia Questionnaire

The subjective feeling of dry mouth was evaluated using the Thai version of the Xerostomia Inventory questionnaire (XI). The XI questionnaire consists of 11 items that can be assigned a score between 1 and 5. A total score ranges from 11 and 55 points, with a total score of ≤ 11 (absence of xerostomia) and > 11 (presence of xerostomia). 25

### Saliva Collection

To evaluate hyposalivation, both unstimulated and stimulated salivary flow tests were conducted. Participants refrained from smoking, eating, or drinking for at least 60 minutes (min) before the assessment. Tests were performed at the same time each day to reduce variations linked to circadian rhythms. The spitting method was used to collect saliva over a 5-minute period. For unstimulated saliva sample collection, participants sat upright to pool saliva and then spat into a collection tube. The salivary flow rate was calculated by dividing the saliva amount by the collection time in milliliters (mL)/min. For stimulated flow, they chewed four to five pieces of paraffin wax for 5 minutes before spitting into another tube. Hyposalivation was defined as an unstimulated salivary flow rate (USSFR) of < 0.1 mL/min and/or a stimulated salivary flow rate (SSFR) of ≤ 0.7 mL/min. 26

### Oral Moisture Assessment

An oral moisture checking instrument (Mucus, Life Co., Ltd.) was used to determine the level of oral moisture. The subjects were asked to rest for around 5 minutes before the measurement. The device's sensor was placed in the center and ∼10 mm (mm) from the tip of the tongue, with a pressure of around 200 g. The measurements were taken in triplicate, and the mean scores were used. Oral moisture levels range from 0 to 99.9, with ≤ 27.9 defining dry mouth, 28.0 to 29.5 defining borderline, and ≥ 29.6 defining normal. 27

### Clinical Oral Dryness Scale

The clinical oral dryness scale (CODS) consists of a 10-point scale, each point indicating an aspect of mouth dryness. 28 The overall CODS was calculated by adding the scores from these 10 characteristics, with the higher scores representing the more severe symptoms.

### Statistical Analysis

All of the statistical analyses were performed by using SPSS version 28 (IBM, Armonk, United States). The descriptive data were summarized using mean, standard deviation (SD), frequency, and percentage as appropriate. The differences between individuals with recurrence periodontitis and those without recurrence periodontitis were compared using the chi-squared test and the student's *t* -test where appropriate. Univariate and multivariate logistic regression were performed to investigate the association between dry mouth (xerostomia and hyposalivation) and recurrent periodontitis among patients undergoing SPT. Variables of interest based on their associations with the previously published studies or with a *p* ≤ 0.2 in univariate analyses were entered into multivariate analyses using a stepwise approach. Odds ratio (OR) and correspondent 95% confidence intervals (95% CI) were calculated. A *p* -value < 0.05 was considered as statistically significant ( *p* < 0.05). Intra-examiner reproducibility was performed before the periodontal examination.

## Results

### Patient Characteristics

**Fig. 1:**
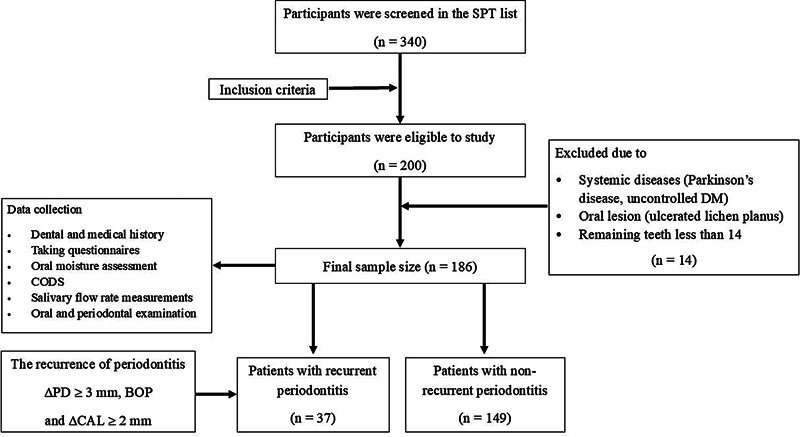
Flow diagram of study participants: A total of 340 patients were identified from the SPT patients list. After reviewing records against the inclusion criteria, 200 patients were deemed eligible for the study. However, 14 participants were excluded due to conditions such as Parkinson's disease, uncontrolled DM, ulcerated lichen planus, and having fewer than 14 teeth. The final study included 186 older adults, with 37 (19.89%) categorized with recurrent periodontitis and 149 (80.11%) without recurrent periodontitis. BOP, bleeding on probing; CAL, clinical attachment level; CODS, clinical oral dryness scale; DM, diabetes mellitus; PD, pocket depth; SPT, supportive periodontal therapy.

Fig. 1 shows the flow chart of participant recruitment. A total of 340 patients were identified from the SPT patients' list, all of whom were scheduled for their regular SPT appointments. After reviewing hospital records against the inclusion criteria, 200 patients were deemed eligible and invited to participate in this study. During the study visit, 14 participants were excluded due to Parkinson's disease, uncontrolled DM, ulcerated lichen planus, and the number of teeth less than 14. Finally, this study included a total of 186 older adults, of which 37 (19.89%) patients were determined to have recurrent periodontitis and 149 (80.11%) were those without any evidence of recurrent periodontitis. There was no significant difference in gender distribution between patients with recurrent periodontitis and those without recurrent periodontitis ( *p* = 0.74). Age ( *p* = 0.79) and BMI ( *p* = 0.44) were similar in both groups. Regarding underlying medical conditions, cardiovascular disease was observed in 67.7% of patients with recurrent periodontitis and 67.8% of those without recurrent periodontitis ( *p* = 0.98). The characteristics of patients in both groups are demonstrated in Table 1 .

**Table 1:** Characteristics of patients with and without recurrence periodontitis ( *n* = 186)

Patient characteristics	Recurrent periodontitis group ( *n* = 37)	Nonrecurrent periodontitis group ( *n* = 149)	*p* -Value
Age (years)	67.78 ± 6.41	68.06 ± 5.3	0.79
Sex			0.74
Males Females	16 (43.2) 21 (56.8)	60 (40.3) 89 (59.7)	
BMI	23.55 ± 3.91	24.53 ± 4.89	0.26 0.44
Normal (18.5–22.9) Underweight (< 18.5) Overweight (> 22.9)	17 (45.9) 1 (2.7) 19 (51.4)	52 (34.9) 7 (4.7) 90 (60.4)	
Medical conditions			
Cardiovascular disease Yes No	25 (67.6) 12 (32.4)	101 (67.8) 48 (32.2)	0.98
Diabetes mellitus Yes No	7 (18.9) 30 (81.1)	29 (19.5) 120 (80.5)	0.94
Osteoporosis Yes No	3 (8.1) 34 (91.9)	22 (14.8) 127 (85.2)	0.42
Number of medications	2 (0, 4)	2 (1, 4)	0.96 0.26
< 5 ≥ 5	29 (78.4) 8 (21.6)	21 (14.1) 128 (85.9)	
Smoking			0.23
Yes No	10 (27) 27 (73)	27 (18.1) 122 (81.9)	
Saliva substitutes			0.79
Yes (at least aid) No	4 (10.8) 33 (89.2)	20 (13.4) 129 (86.6)	
Frequency of tooth brushing			0.35
< 2 ≥ 2	0 37 (100)	7 (4.7) 142 (95.3)	
Interdental cleaning			0.92
Not used Some days Everyday	3 (8.1) 19 (51.4) 15 (40.5)	10 (6.7) 74 (49.7) 65 (43.6)	
Barthel ADL index			N/A
> 12 (independence)	37 (100)	149 (100)	
GDS	2 (1, 3)	2 (1, 3)	0.99 0.53
Normal (0–4) Some degree (5–9)	30 (81.1) 7 (18.9)	127 (85.2) 22 (14.8)	
Number of remaining teeth	22.57 ± 4.17	22.54 ± 4.5	0.98 0.71
< 20 20–32	10 (27) 27 (73)	45 (30.2) 104 (69.8)	
Number of occlusal pairings	4.35 ± 2.4	4.11 ± 2.42	0.58 0.62
< 4 ≥ 4	13 (35.1) 24 (64.9)	59 (39.6) 90 (60.4)	
Denture wearing			0.98
Yes No	24 (64.9) 13 (35.1)	97 (65.1) 52 (34.9)	
Xerostomia Inventory Questionnaire (XI)	21.51 ± 6.62	21.83 ± 6.65	0.79 0.42
≤ 11 (absence of xerostomia) > 11 (presence of xerostomia)	3 (8.1) 34 (91.9)	7 (4.7) 142 (95.3)	
Hyposalivation (USSFR < 0.1 mL/min and/or SSFR ≤ 0.7 mL/min)			0.02 a
Yes No	13 (35.1) 24 (64.9)	24 (16.1) 125 (83.9)	
Salivary flow rate			
USSFR (< 0.1 mL/min) Yes No	0.38 ± 0.26 9 (24.3) 28 (75.7)	0.53 ± 0.35 11 (7.4) 138 (92.6)	0.01 a 0.006 a
SSFR (≤ 0.7 mL/min) Yes No	1.31 ± 0.66 7 (18.9) 30 (81.1)	1.44 ± 0.72 16 (10.7) 133 (89.3)	0.30 0.17
Oral moisture checking device	27.82 ± 1.73	27.41 ± 1.74	0.20 0.12
Dry mouth (≤ 27.9) Borderline (28–29.5) Normal (≥ 29.6)	16 (43.2) 18 (48.6) 3 (8.1)	90 (60.4) 46 (30.9) 13 (8.7)	
Total CODS	2 (1, 3)	1 (0, 2)	0.01 a

Abbreviations: ADL, activity daily living; BMI, body mass index; CODS, clinical oral dryness scale; GDS, geriatric depression scale; SSFR, stimulated salivary flow rate; USSFR, unstimulated salivary flow rate; XI, xerostomia inventory.

Note: Mean ± SD, median (IQR1, IQR3), or number (%) is presented.

a *p* < 0.05.

### Xerostomia, Hyposalivation, and Oral Moisture

A total of 176 of 186 patients had xerostomia, of whom 34 (91.9%) and 142 (95.3%) were in the recurrent periodontitis and nonrecurrent periodontitis group. There was no significant association between the presence/absence of xerostomia and recurrent periodontitis ( *p* = 0.42, Table 1 ). Interestingly, hyposalivation was significantly more predominant among participants with recurrent periodontitis than those without (35.1 vs. 16.1%, respectively) ( *p* = 0.02). When focusing on salivary flow rate, it was found that the mean of USSFR in the recurrent periodontitis group was statistically significantly lower than in those without recurrent periodontitis (0.38 vs. 0.53 mL/min, *p* = 0.01, Table 1 ). There was also a significant association between USSFR < 0.1 mL/min and recurrent periodontitis ( *p* = 0.006). Nevertheless, for SSFR, no statistically significant difference was observed between the two groups ( *p* = 0.17). The distribution of oral moisture levels was not significantly different between those with and without recurrent periodontitis ( *p* = 0.12). Moreover, the median CODS observed in the recurrent periodontitis group was significantly higher than in the nonrecurrent periodontitis group (2 vs. 1, *p* = 0.01, Table 1 ).

### Periodontal Status and SPT Interval

The results showed a statistically significant difference between the two groups on the %BOP ( *p* < 0.001), PD = 3 to 4 mm ( *p* = 0.001), PD ≥ 5 mm ( *p* < 0.001), CAL ≥ 5 mm ( *p* = 0.003). However, the percentage of sites with PD = 1 to 2 mm was statistically significantly lower in those with recurrent periodontitis ( *p* = 0.001). The number of third-degree mobility teeth was significantly higher in the recurrent periodontitis group than in those without recurrent periodontitis ( *p* = 0.001). With respect to the percentage of plaque score, CAL (0–2 mm), CAL (3–4 mm), first-degree and second-degree mobility of teeth, and SPT intervals, no statistically significant difference was observed between these two groups ( Table 2 ).

**Table 2:** Clinical periodontal parameters and SPT interval of participants ( *n* = 186)

Variable	Recurrent periodontitis group ( *n* = 37)	Nonrecurrent periodontitis group ( *n* = 149)	*p* -Value
**%** Plaque score	50.72 ± 21.80	43.86 ± 19.49	0.06
**%** BOP	45.77 ± 20.52	32.87 ± 17.54	< 0.001 a
PD			
% site PD = 1–2 mm % site PD = 3–4 mm % site PD ≥ 5 mm	48.15 ± 17.5 48.38 ± 14.61 2.5 (1.17, 6)	62.36 ± 17.39 36.84 ± 16.82 0 (0, 0.78)	0.001 b 0.001 b < 0.001 a
CAL			
% site CAL = 0–2 mm % site CAL = 3–4 mm % site CAL ≥ 5 mm	23.48 ± 17.58 49.22 ± 13.87 27.19 ± 19.24	28.53 ± 18.53 51.75 ± 11.91 18.34 ± 15.40	0.14 0.27 0.003 b
**Tooth mobility**			
Number of tooth mobility % 1° mobility tooth % 2° mobility tooth % 3° mobility tooth	0 (0, 4.5) 0 (0, 11.33) 0 (0, 4.2) 0 (0, 1.92)	0 (0, 2) 0 (0, 10.44) 0 (0, 0) 0 (0, 0)	0.53 0.80 0.05 0.001 b
**SPT interval**	9 (6, 13.5)	8 (5, 12.5)	0.21 0.20
3 mo > 3 mo ≤ 6 mo > 6 mo	1 (2.7) 9 (24.3) 27 (73)	19 (12.8) 35 (23.5) 95 (63.8)	

Abbreviations: BOP, bleeding on probing; CAL, clinical attachment level; PD, pocket depth; SPT, supportive periodontal therapy.

Note: Mean ± SD, median (IQR1, IQR3), or number (%) is presented.

a *p* < 0.001.

b *p* < 0.05.

### Periodontal Parameters in Patients with and without Xerostomia and Hyposalivation

Table 3 shows the periodontal parameters in individuals with and without xerostomia and hyposalivation. The mean percentage of sites with ΔCAL = 1 mm among patients with xerostomia was significantly lower than in those without xerostomia ( *p* = 0.01). However, for other periodontal parameters, including the percentage of sites with PD = 4 mm, sites with PD 5 mm, sites with CAL = 2 mm., and sites with CAL = 3 mm, no significant difference was found between xerostomia and non-xerostomia patients ( Table 3 ). Regarding hyposalivation, this study demonstrated that 37 out of 186 (19.89%) had hyposalivation. It was found that there was a statistically significant difference in the mean percentage of sites with PD ≥ 5 mm between the two groups ( *p* = 0.002), with the mean percentage of sites with PD ≥ 5 mm in the hyposalivation group being significantly higher than the non-hyposalivation group ( Table 3 ). Of note, the mean percentage of sites with ΔCAL = 2 was significantly lower in patients with hyposalivation than in individuals without hyposalivation ( *p* = 0.02).

**Table 3:** Periodontal parameters in patients with and without xerostomia and hyposalivation ( *n* = 186)

Periodontal parameters	Xerostomia		*p* -Value	Hyposalivation		*p* -Value
	Yes ( *n* = 176)	No ( *n* = 10)		Yes ( *n* = 37)	No ( *n* = 149)	
% site with PD = 4 mm	4.79 ± 4.2	3.36 ± 5.42	0.84	5.23 ± 5.6	4.59 ± 3.88	0.06
% site with PD ≥ 5 mm	1.33 ± 2.55	1.79 ± 3.6	0.23	2.14 ± 3.98	1.16 ± 2.11	0.002 a
% site with ΔCAL = 1 mm	23.48 ± 9.74	26.78 ± 4.75	0.01 a	26.58 ± 9.97	22.93 ± 9.35	0.68
% site with ΔCAL = 2 mm	10.31 ± 7.71	11.43 ± 7.46	0.73	9.29 ± 5.85	10.64 ± 8.06	0.02 a
% site with ΔCAL = 3 mm	3.42 ± 3.67	4.85 ± 5.39	0.15	3.99 ± 3.62	3.38 ± 3.82	0.94

Abbreviations: CAL, clinical attachment level; PD, pocket depth.

Note: Mean ± SD are presented.

a *p* < 0.05.

### The Association between Hyposalivation and Recurrent Periodontitis

Univariate and multivariate logistic regression models for recurrent periodontitis, adjusted for all potential confounding factors, are shown in Table 4 . In univariate analysis, hyposalivation was found to be associated with a recurrence of periodontitis during the SPT program (OR = 2.82, 95% CI: 1.26–6.30, *p* = 0.01). Moreover, the %BOP was also significantly associated with recurrent periodontitis in patients who underwent SPT (OR = 1.04, 95% CI: 1.02–1.06, *p* < 0.001). After adjusting for potential confounding factors (age, gender, smoking, GDS, number of medications, SPT interval, number of occlusal pairings, the percentage of plaque score, and the %BOP) in multivariate analysis, it was found that both hyposalivation (OR = 2.63, 95 CI%: 1.05–6.58, *p* = 0.04 and OR = 1.04, 95 CI%: 1.02–1.07, *p* = 0.001) and the %BOP remained statistically significant with recurrence of periodontitis.

**Table 4:** Univariate and multivariate logistic regression analysis of the association between hyposalivation and recurrence periodontitis ( *n* = 186)

Variables	Recurrent periodontitis ( *n* = 37)	Nonrecurrent periodontitis ( *n* = 149)	Univariate analysis		Multivariate analysis	
			Unadjusted OR (95% CI)	*p-* Value	Adjusted OR (95% CI)	*p-* Value
Hyposalivation						0.04 a
No Yes	24 (64.9) 13 (35.1)	125 (83.9) 24 (16.1)	1 2.82 (1.26–6.30)	0.011 a	1 2.63 (1.05–6.58)	
Age, years	67.78 ± 6.41	68.06 ± 5.3	0.99 (0.93–1.06)	0.78	0.99 (0.92–1.06)	0.74
Gender						0.47
Males Females	16 (43.2) 21 (56.8)	60 (40.3) 89 (59.7)	1 0.89 (0.43–1.83)	0.74	1 1.47 (0.52–4.18)	
Smoking						0.12
No Yes	27 (73) 10 (27)	122 (81.9) 27 (18.1)	1 1.67 (0.73–3.86)	0.23	1 2.59 (0.79–8.5)	
GDS						0.33
Normal Some degree	30 (81.1) 7 (18.9)	127 (85.2) 22 (14.8)	1 1.35 (0.53–3.45)	0.53	1 1.73 (0.57–5.28)	
Number of medications						0.88
< 5 ≥ 5	29 (78.4) 8 (21.6)	21 (14.1) 128 (85.9)	1 1.68 (0.68–4.17)	0.26	1 1.09 (0.39–3.03)	
SPT interval (months)						0.15 0.13
3 > 3 ≤6 > 6	1 (2.7) 9 (24.3) 27 (73)	19 (12.8) 35 (23.5) 95 (63.8)	1 4.89 (0.58–41.53) 5.4 (0.69–42.19)	0.15 0.12	1 5.3 (0.55–51.54) 5.44 (0.63–47.24)	
Number of occlusal pairings						0.49
< 4 ≥ 4	13 (35.1) 24 (64.9)	59 (39.6) 90 (60.4)	1 1.21 (0.57–2.56)	0.62	1 1.35 (0.59–3.09)	
% Plaque score	50.72 ± 21.80	43.86 ± 19.49	1.02 (1–1.037)	0.07	0.99 (0.07–1.02)	0.46
% BOP	45.77 ± 20.52	32.87 ± 17.54	1.04 (1.02–1.06)	< 0.001 b	1.04 (1.02–1.07)	0.001 a

Abbreviations: BOP, bleeding on probing; CI, confidence interval; GDS, geriatric depression scale; OR, odd ratio; SPT, supportive periodontal therapy; USSFR, unstimulated salivary flow rate.

Note: Mean ± SD, number (%) are presented.

a *p* < 0.05.

b *p* < 0.001.

## Discussion

The results of this study indicate that hyposalivation is significantly associated with an approximately twofold increase in the risk of periodontitis recurrence among older adults during SPT, whereas xerostomia shows no association. This study represents the first investigation of this relationship. When compared with similar studies by Sparrow et al., discrepancies were noted, potentially arising from variations in the age demographics of the participants. The previous study included adults aged 34 to 79 years, with a mean age of 61 years, whereas the current study focused on older adults, with a mean age of 68.01 years. 16 They aimed to explore the relationship between the clinical outcomes of periodontal treatment and patients presenting with hyposalivation. The chi-square test was used, while our study ultimately applied multivariate logistic regression analysis. Thus, this study offers the chance to assess the likelihood of periodontitis recurrence among individuals with hyposalivation, considering additional relevant variables.

Concerning the mean USSFR, individuals with recurrent periodontitis demonstrated a significantly lower rate compared with those without the condition. There exists a substantial correlation between a USSFR and the occurrence of recurrent periodontitis, while the differences in SSFR were not statistically significant. These findings are consistent with previous research indicating that a deficiency in saliva may impair self-cleansing mechanisms, diminish the protective properties of saliva, hinder lubrication, and exacerbate dehydration. Furthermore, insufficient salivary flow may adversely affect the resilience and viscoelasticity of the fluid surrounding the periodontium. 11 13 14 29 Moreover, this could be attributed to the physiological functions of the submandibular gland, which is the major source of unstimulated salivary flow. It produces plenty of mucin, which coats the oral tissues, offering lubrication, attenuating colonization of *Aggregatibacter actinomycetemcomitans* , and protecting oral moisture. 29 Conversely, during stimulated salivary flow function, the secretion of saliva from the parotid gland experiences a significant increase, generating abundant buffered saliva. 30 Therefore, the older population is more likely to be susceptible to inflammation, especially among older adults, where an increased incidence of dry mouth and periodontitis is seen.

Nevertheless, the present study used distinct categorical criteria for identifying the recurrence of periodontitis, specifically an increase in PD ≥ 3 mm accompanied by persistent BOP and an increase in CAL ≥ 2 mm. These criteria differ from those utilized in previous studies, such as the criteria applied by Cortellini et al., which involved a change in PD ≥ 2 mm with persistent BOP and a change in CAL ≥ 2 mm 23 or studies that only focused on CAL, such as the study conducted by Lang et al. and Waithongkam et al. (ΔCAL ≥ 2 mm), 31 32 Beck (ΔCAL ≥ 3 mm.), 33 and Heredia-P et al. (ΔCAL > 2 mm). 34 We hypothesized that the accumulation of disease over time and the natural aging process of the periodontium, which displays thinning of the oral epithelium, decreased keratinization, and the reduction or loss of periodontal ligament and connective tissue elasticity, 35 might have occurred prior to the onset of periodontitis and contributed to its recurrence. Therefore, the observed enhancement in PD alteration at a depth of 3 mm was examined as an indicative of recurrent periodontitis in older adults.

The results of the periodontal status in both groups indicated that the intensity of inflammation, as measured by BOP, PD, and CAL, was higher in the recurrent group. However, both groups had no statistically significant differences in plaque scores, which suggested that participants were comparable in control of their oral hygiene. For SPT interval, the occurrence of recurrent periodontitis was increased within the group of time intervals, which were > 3-month group, ≤ 6-month group, and > 6-month group, but no significant difference. This might be the result of SPT adherence in the suggested time. Similar to previous studies, the different SPT visits demonstrated no statistically significant difference in periodontal health in individuals who received SPT regularly. 8 However, Rosén et al. suggested that recall intervals of up to a year would be acceptable to slow down the progression of periodontal disease in low-risk patients. 36

This study demonstrated a statistically significant difference in the mean proportion of sites with PD ≥ 5 mm, which was substantially higher in the hyposalivation group compared with the non-hyposalivation group. The link between hyposalivation and periodontal disease was consistent with a previous study that characterized a reduction in self-cleansing, antimicrobial activity, and mucosal integrity, resulting in changes in the composition of microbial plaque. 37 Consequently, it enhances the probability of developing periodontal disease. 31 35 This part showed that hyposalivation was related to inflammation rather than altered physiology in the elderly population.

The study also examined the associations between factors that might contribute to the development of dry mouth, such as female sex, smoking, and mouth breathing by univariate and multivariate logistic regression models. 33 However, our study found no significant effect of these factors on periodontal conditions; it was found that both hyposalivation and BOP remained statistically significant with the recurrence of periodontitis. Medications commonly prescribed to elderly patients could impact salivary flow rate, but our study did not find it to be a factor affecting periodontal status. 38 In future investigations, it would be advantageous to consider both the dosage and duration of medications administered.

The limitation of this research is the participant selection process, which primarily involved only one group of older adults who were either physically healthy or cooperative with appointments. Hence, it may be necessary to investigate this trend in further populations that encompass broader demographic characteristics, and we should conduct the study among older adults who are at risk of dry mouth, such as those taking anticholinergic or antihypertensive medications.

## Conclusion

The results of this study demonstrated that hyposalivation was associated with an increased occurrence of recurrent periodontitis. Regular periodontal care, comprising a comprehensive examination along with recommendations for managing dry mouth, can enable elderly people with periodontitis to preserve their oral health. The maintenance of oral mucosal moisture can potentially prevent the occurrence of dry mouth and the subsequent reoccurrence of periodontitis.

## References

[1] Hajishengallis G Chavakis T Lambris J D Current understanding of periodontal disease pathogenesis and targets for host-modulation therapy Periodontol 2000 2020 84 01 14 34 32844416 10.1111/prd.12331 PMC7457922

[2] Herrero E R Fernandes S Verspecht T Dysbiotic biofilms deregulate the periodontal inflammatory response J Dent Res 2018 97 05 547 555 29394879 10.1177/0022034517752675

[3] Chen M X Zhong Y J Dong Q Q Wong H M Wen Y F Global, regional, and national burden of severe periodontitis, 1990-2019: an analysis of the Global Burden of Disease Study 2019 J Clin Periodontol 2021 48 09 1165 1188 34101223 10.1111/jcpe.13506

[4] López-Otín C Blasco M A Partridge L Serrano M Kroemer G The hallmarks of aging Cell 2013 153 06 1194 1217 23746838 10.1016/j.cell.2013.05.039 PMC3836174

[5] Darby I Risk factors for periodontitis & peri-implantitis Periodontol 2000 2022 90 01 9 12 35913624 10.1111/prd.12447 PMC9804916

[6] Manresa C Sanz-Miralles E C Twigg J Bravo M Supportive periodontal therapy (SPT) for maintaining the dentition in adults treated for periodontitis Cochrane Database Syst Rev 2018 1 01 CD009376 29291254 10.1002/14651858.CD009376.pub2 PMC6491071

[7] Lorentz T CM Cota L OM Cortelli J R Vargas A MD Costa F O Prospective study of complier individuals under periodontal maintenance therapy: analysis of clinical periodontal parameters, risk predictors and the progression of periodontitis J Clin Periodontol 2009 36 01 58 67 19017035 10.1111/j.1600-051X.2008.01342.x

[8] Axelsson P Lindhe J The significance of maintenance care in the treatment of periodontal disease J Clin Periodontol 1981 8 04 281 294 6947992 10.1111/j.1600-051x.1981.tb02039.x

[9] Lang N P Tonetti M S Periodontal risk assessment (PRA) for patients in supportive periodontal therapy (SPT) Oral Health Prev Dent 2003 1 01 7 16 15643744

[10] Tanasiewicz M Hildebrandt T Obersztyn I Xerostomia of various etiologies: a review of the literature Adv Clin Exp Med 2016 25 01 199 206 26935515 10.17219/acem/29375

[11] Gupta A Epstein J B Sroussi H Hyposalivation in elderly patients J Can Dent Assoc 2006 72 09 841 846 17109806

[12] Ouanounou A Xerostomia in the geriatric patient: causes, oral manifestations, and treatment Compend Contin Educ Dent 2016 37 05 306 311, quiz 312 27213776

[13] Dawes C Salivary flow patterns and the health of hard and soft oral tissues J Am Dent Assoc 2008 139(Suppl):18S 24S 18460676 10.14219/jada.archive.2008.0351

[14] Lamster I B Asadourian L Del Carmen T Friedman P K The aging mouth: differentiating normal aging from disease Periodontol 2000 2016 72 01 96 107 27501493 10.1111/prd.12131

[15] Hirotomi T Yoshihara A Ogawa H Ito K Igarashi A Miyazaki H A preliminary study on the relationship between stimulated saliva and periodontal conditions in community-dwelling elderly people J Dent 2006 34 09 692 698 16473454 10.1016/j.jdent.2006.01.001

[16] Sparrow T V Fritz P C Sullivan P J Ward W E Regular maintenance appointments after non-surgical scaling and root planing support periodontal health in patients with or without dry mouth: a retrospective study Clin Exp Dent Res 2021 7 05 647 655 33474841 10.1002/cre2.401 PMC8543481

[17] Turner M D Ship J A Dry mouth and its effects on the oral health of elderly people J Am Dent Assoc 2007 138(Suppl):15S 20S 17761841 10.14219/jada.archive.2007.0358

[18] Caton J G Armitage G Berglundh T A new classification scheme for periodontal and peri-implant diseases and conditions - introduction and key changes from the 1999 classification J Clin Periodontol 2018 45 20 S1 S8 29926489 10.1111/jcpe.12935

[19] Oliveira Costa F Miranda Cota L O Pereira Lages E J Progression of periodontitis in a sample of regular and irregular compliers under maintenance therapy: a 3-year follow-up study J Periodontol 2011 82 09 1279 1287 21342000 10.1902/jop.2011.100664

[20] Costa F O Cota L OM Cortelli J R Surgical and non-surgical procedures associated with recurrence of periodontitis in periodontal maintenance therapy: 5-year prospective study PLoS One 2015 10 10 e0140847 26496187 10.1371/journal.pone.0140847 PMC4619675

[21] Mahoney F I Barthel D W Functional evaluation: the Barthel index Md State Med J 1965 14 61 65 14258950

[22] Sheikh J I Geriatric Depression Scale (GDS): recent evidence and development of a shorter version New York Haworth Press 1986 165

[23] Cortellini P Buti J Pini Prato G Tonetti M S Periodontal regeneration compared with access flap surgery in human intra-bony defects 20-year follow-up of a randomized clinical trial: tooth retention, periodontitis recurrence and costs J Clin Periodontol 2017 44 01 58 66 27736011 10.1111/jcpe.12638

[24] Listgarten M A Levin S Positive correlation between the proportions of subgingival spirochetes and motile bacteria and susceptibility of human subjects to periodontal deterioration J Clin Periodontol 1981 8 02 122 138 6941979 10.1111/j.1600-051x.1981.tb02352.x

[25] Kasa K Worakajit P Sinsen S Samnieng P Translation, validation and reliability of a Thai version of the xerostomia inventory[article in Thai]SWU Dent J. 2022 15 10 20

[26] University of Southern California School of Dentistry Navazesh M Kumar S KS Measuring salivary flow: challenges and opportunities J Am Dent Assoc 2008 139(Suppl):35S 40S 18460678 10.14219/jada.archive.2008.0353

[27] Fukushima Y Yoda T Araki R Evaluation of oral wetness using an improved moisture-checking device for the diagnosis of dry mouth Oral Sci Int 2017 14 33 36

[28] Challacombe S J Osailan S M Proctor G B Clinical scoring scales for assessment of dry mouth Springer Berlin Heidelberg 2015 119 132

[29] Giannobile W V Beikler T Kinney J S Ramseier C A Morelli T Wong D T Saliva as a diagnostic tool for periodontal disease: current state and future directions Periodontol 2000 2009 50 52 64 19388953 10.1111/j.1600-0757.2008.00288.x PMC5695225

[30] Dodds M W Johnson D A Yeh C K Health benefits of saliva: a review J Dent 2005 33 03 223 233 15725522 10.1016/j.jdent.2004.10.009

[31] Lang N P Joss A Orsanic T Gusberti F A Siegrist B E Bleeding on probing. A predictor for the progression of periodontal disease? J Clin Periodontol 1986 13 06 590 596 3489010 10.1111/j.1600-051x.1986.tb00852.x

[32] Waithongkam A LP Teparat-Burana T Patient accessibility to supportive periodontal therapy and the occurrence of recurrent periodontitis: a retrospective study in the Faculty of Dentistry, Mahidol University Mo Dent J 2022 42 109 118

[33] Beck J D Methods of assessing risk for periodontitis and developing multifactorial models J Periodontol 1994 65 468 478 10.1902/jop.1994.65.5s.468 8046563

[34] Heredia-P A M Lafaurie G I Bautista-Molano W Predictive factors related to the progression of periodontal disease in patients with early rheumatoid arthritis: a cohort study BMC Oral Health 2019 19 01 240 31703715 10.1186/s12903-019-0939-6 PMC6842164

[35] Bhadbhade S Aging & periodontium Int J Dent Oral Sci 2015 2 79 83

[36] Rosén B Olavi G Badersten A Rönström A Söderholm G Egelberg J Effect of different frequencies of preventive maintenance treatment on periodontal conditions. 5-Year observations in general dentistry patients J Clin Periodontol 1999 26 04 225 233 10223393 10.1034/j.1600-051x.1999.260405.x

[37] Villa A Polimeni A Strohmenger L Cicciù D Gherlone E Abati S Dental patients' self-reports of xerostomia and associated risk factors J Am Dent Assoc 2011 142 07 811 816 21719803 10.14219/jada.archive.2011.0269

[38] Singh M L Papas A Oral implications of polypharmacy in the elderly Dent Clin North Am 2014 58 04 783 796 25201542 10.1016/j.cden.2014.07.004

