# Supplementary material for: The Association between Dry Mouth and the Periodontal Status in Older Adults Undergoing Supportive Periodontal Therapy
Source: Eur J Dent. 2025 May 21;20(1):226–35. doi: 10.1055/s-0045-1809183 (PMC12890395; doi:10.1055/s-0045-1809183)
Supplement: Supplementary file 1 — Supplementary Material [file 10-1055-s-0045-1809183-s24124011.pdf]

Supplementary Table S1 Geriatric Depression Scale (GDS-15)

Symptoms	Yes	No
1) Are you basically satisfied with your life?		
2) Have you dropped many of your activities and interests?		
3) Do you feel that your life is empty?		
4) Do you often get bored?		
5) Are you in good spirits most of the time?		
6) Are you afraid that something bad is going to happen to you		
7) Do you feel happy most of the time?		
8) Do you often feel helpless?		
9) Do you prefer to stay at home, rather than going out and doing new things?		
10) Do you feel you have more problems with memory than most?		
11) Do you think it is wonderful to be alive now?		
12) Do you feel pretty worthless the way you are now?		
13) Do you feel full of energy?		
14) Do you feel that your situation is hopeless?		
15) Do you think that most people are better off than you are?		

 © 2025. The Author(s).
